# Multiparametric MRI to quantify disease and treatment response in mice with myeloproliferative neoplasms

**DOI:** 10.1172/jci.insight.161457

**Published:** 2022-10-10

**Authors:** Tanner H. Robison, Manisha Solipuram, Kevin Heist, Ghoncheh Amouzandeh, Winston Y. Lee, Brock A. Humphries, Johanna M. Buschhaus, Avinash Bevoor, Anne Zhang, Kathryn E. Luker, Kristen Pettit, Moshe Talpaz, Dariya Malyarenko, Thomas L. Chenevert, Brian D. Ross, Gary D. Luker

**Affiliations:** 1 Department of Radiology and Center for Molecular Imaging;; 2 Department of Biomedical Engineering;; 3 Department of Pathology;; 4 Department of Internal Medicine, Division of Hematology and Oncology;; 5 Department of Biological Chemistry; and; 6 Department of Microbiology and Immunology, University of Michigan, Ann Arbor, Michigan, USA.

**Keywords:** Bone Biology, Oncology, Bone disease, Bone marrow, Diagnostic imaging

## Abstract

Histopathology, the standard method to assess BM in hematologic malignancies such as myeloproliferative neoplasms (MPNs), suffers from notable limitations in both research and clinical settings. BM biopsies in patients fail to detect disease heterogeneity, may yield a nondiagnostic sample, and cannot be repeated frequently in clinical oncology. Endpoint histopathology precludes monitoring disease progression and response to therapy in the same mouse over time, missing likely variations among mice. To overcome these shortcomings, we used MRI to measure changes in cellularity, macromolecular constituents, and fat versus hematopoietic cells in BM using diffusion-weighted imaging (DWI), magnetization transfer, and chemical shift–encoded fat imaging. Combining metrics from these imaging parameters revealed dynamic alterations in BM following myeloablative radiation and transplantation. In a mouse MPL^W515L^ BM transplant model of MPN, MRI detected effects of a JAK2 inhibitor, ruxolitinib, within 5 days of initiating treatment and identified differing kinetics of treatment responses in subregions of the tibia. Histopathology validated the MRI results for BM composition and heterogeneity. Anatomic MRI scans also showed reductions in spleen volume during treatment. These findings establish an innovative, clinically translatable MRI approach to quantify spatial and temporal changes in BM in MPN.

## Introduction

BM, one of the largest organs in the body, consists of heterogeneous, distinct microenvironments in different bones and regions within a bone. Heterogeneity of BM compartments (1) throughout the body regulates changes in hematopoiesis during aging, responses to interventions such as BM transplant, and manifestations of hematologic and metastatic cancers. Despite critical functions of BM in normal physiology and disease, direct, quantitative assessment of BM heterogeneity, composition, and architecture over time remains an unmet need in research and clinical settings. Investigators and clinicians rely on alternative metrics, such as multiparametric flow cytometry, cytogenetics, molecular analysis, and cell counts of peripheral blood to evaluate and stage disease status (2–5). Disease-specific secondary metrics like spleen volume and constitutional symptoms provide additional measures of severity for many hematologic diseases. However, these approaches fail to capture spatial heterogeneity in BM environments in health, disease, and therapy over time.

Myeloproliferative neoplasms (MPNs), a class of blood cancers, typically arise from mutations in the thrombopoietin receptor (MPL), JAK2, or calreticulin that constitutively activate JAK/STAT signaling in hematopoietic stem and progenitor cells (HSPCs) (6–8). Unregulated JAK/STAT signaling leads to clonal expansion of malignant cells in BM and overproduction of leukocytes, red blood cells, and/or platelets (9). MPNs include essential thrombocythemia (excess platelets), polycythemia vera (excess red blood cells), and myelofibrosis (MF). MF, defined by abnormalities in production of blood cells and deposition of extracellular matrix fibers in BM, may occur as a primary malignancy or secondary to other MPNs. In MF, cytokines produced by abnormal megakaryocytes, cells that generate platelets, create an inflammatory BM environment that stimulates progression from prefibrotic disease to advanced BM fibrosis (10–14). BM dysregulation and fibrosis are directly implicated in migration of HSPCs to sites of extramedullary hematopoiesis, resulting in hepatosplenomegaly (15, 16). Given that aberrant JAK/STAT signaling occurs in almost all cases of MF, inhibitors of JAK2 have become the primary treatment approach for patients. JAK2 inhibitors reduce constitutional symptoms and splenomegaly, and reductions in total symptom scores and spleen volume remain standard endpoints in clinical trials (17, 18). However, current JAK2 inhibitors do not robustly reduce BM fibrosis, restore normal BM cellularity and macroscale architecture, or eliminate malignant HSPCs. Drug companies now are focusing on developing drugs that reverse these hallmark features of MF (19). While molecular techniques can measure mutant allele frequencies in HSPCs, there is a clear, unmet need for quantitative methods to noninvasively analyze BM cellularity and architecture in living subjects over time.

Histology in preclinical models and biopsy in patients allow direct assessment of BM architecture and composition. Harvesting bones for histology in preclinical models precludes serial studies of the same animal during disease progression and therapy, and investigators typically analyze only a limited number of histologic sections from a bone. Biopsies in patients sample a very small percentage of total BM from a single anatomic site, the iliac crest. Although regarded as the gold standard for evaluating BM, biopsy suffers from notable deficiencies: a) it is an invasive, painful procedure; b) it offers limited evaluation of heterogeneity; c) it poses the potential for a nondiagnostic sample; and d) its semiquantitative analysis is subject to inter-reader variations (20–22). These deficiencies reduce the value of BM composition and architecture and its changes over time as metrics for disease staging and treatment monitoring.

While monitoring mutant HSPCs through ex vivo molecular studies is vital to treatment of MF and other MPNs, successful therapy requires restoration of normal cellularity and architecture, including reversal of fibrosis, throughout hematopoietically active BM. Achieving these results requires better preclinical and ultimately clinical methods to evaluate BM in living subjects. Imaging offers an exciting potential to noninvasively analyze BM and its heterogeneity over time. However, imaging rarely has been used to investigate key BM manifestations of disease in preclinical models or even in routine clinical practice. Reasons for limited applications of BM imaging include failure to optimize parameters for anticipated pathologic changes and reliance on qualitative, rather than quantitative, readouts. Imaging may be particularly informative for MPNs, where dysregulated expansion of hematopoietic cells replaces normal fat and in the case of MF, progressive fibrosis that disrupts normal BM architecture.

Toward the goal of developing a quantitative imaging method for MF, our group previously published results using quantitative MRI for BM fat in a small series of patients (23). The study established feasibility of this quantitative MRI technique to detect early changes in BM during treatment with a clinically approved JAK2 inhibitor, ruxolitinib. Mouse models of MF present an opportunity to evaluate MRI for noninvasive detection of disease-associated heterogeneity in BM with histological truth for validation. Mouse models also allow us to test and validate additional MRI parameters to analyze cellularity and fibrosis in BM. While investigators commonly use mouse models to investigate MF and other MPNs, past research documents relevant differences between human and mouse BM. Hematopoietically active BM distributes throughout the entire skeleton in infants with progressive expansion of BM fat from distal to proximal over time. By age 25, adult humans have active BM only in the axial skeleton (spine, sternum, pelvis, and skull) (24–27). Within a human long bone such as the femur, BM fat increases first in epiphyses, followed by the mid-diaphysis and then expanding to the remainder of the bone (25, 27). Mice show relatively less BM fat than humans, although fat appears in the distal tibia by 4 weeks and increases progressively to fill the entire marrow space of the distal tibia by 8 weeks of age (28). In mice, hematopoietically active BM remains in both trabecular bone and the medullary space. Relative to patients, MRI of mouse BM presents greater technical challenges because the small BM volumes in mice require high spatial resolution. Despite these challenges, 1 prior study using a Gata-1^lo^ MF mouse model and single-parameter MRI showed qualitative changes associated with BM inflammation during disease progression (29).

In this study, we demonstrate that quantitative MRI sequences, selected to identify key pathologic features of MPNs overall with a focus on MF, can longitudinally quantify signal changes associated with progression from healthy to hypercellular BM with splenomegaly in living mice. Importantly, we found that quantitative MRI detects reversion of BM changes in response to ruxolitinib treatment. These data establish a new MRI-based approach to noninvasively investigate disease status and response to therapy in preclinical models of MPNs and MF. Furthermore, as the quantitative MRI approaches investigated in mice are readily translatable to clinical MRI, this study sets the stage to investigate quantitative, multiparametric MRI for image-based biomarkers to complement and advance current clinical metrics to assess disease status in patients with MF.

## Results

### Quantitative MRI metrics capture heterogeneity along length of mouse tibia.

**Figure 1:**
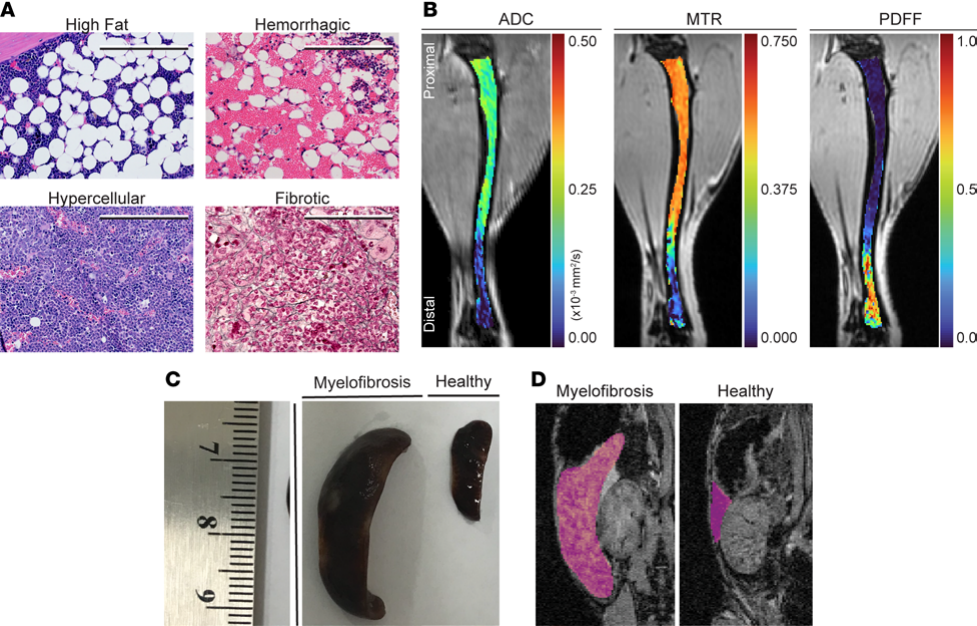
Quantitative MRI metrics — ADC, PDFF, and MTR — selected to analyze BM pathology. (**A**) Histology of observed BM pathologies in an MPL^W515L^ transplant mouse model of MF. High fat, hemorrhagic, and hypercellular panels are stained with H&E, while reticulin staining shows fibrosis. Scale bar: 100 μm. (**B**) Representative images for quantified ADC, MTR, and PDFF MRI parameters in the tibia of a healthy BALB/c mouse. Images display values for each parameter on a pseudocolor scale with red and blue marking high and low values, respectively. Note different scales for each imaging parameter. (**C** and **D**) Qualitative differences in spleen size and MRI volume of a healthy mouse and a mouse with progressive MPN/MF. We manually segmented spleens (outlined with pink overlay) for volume measurements. Diseased spleens imaged/harvested 36 days after disease initiation with MPL^W515L^ transduction-transplantation mouse model.

To quantify distinct disease pathologies of MPN/MF in BM (Figure 1A), we selected 3 clinically approved quantitative MRI metrics: apparent diffusion coefficient (ADC), magnetization transfer ratio (MTR), and proton density fat fraction (PDFF) (Figure 1B) to image BM. ADC quantifies the mobility of water (30), which is impeded by cell membranes, fat, and subcellular constituents. ADC is considered to reflect tissue cell density in solid tumors. We hypothesized ADC would be sensitive to changes in BM cellularity and fat during MPN progression and possibly reveal early reversal of hypercellularity during efficacious therapy. MTR detects the exchange of magnetization between water (detectable MRI signal) molecules and the solid macromolecular matrix (undetectable MRI signal) (31, 32). We hypothesized that progressive fibrosis in MF would increase the MTR value. We expected that PDFF, which quantifies the relative fraction of fat versus water signal based on known differences in chemical shift of protons in each environment (33, 34), would decrease with increasing expansion of hematopoietic cells in BM during disease progression. We further wanted to quantify variations in spleen size by MRI (Figure 1, C and D), which is done routinely in the clinic, to provide an additional clinical correlate of disease.

**Figure 2:**
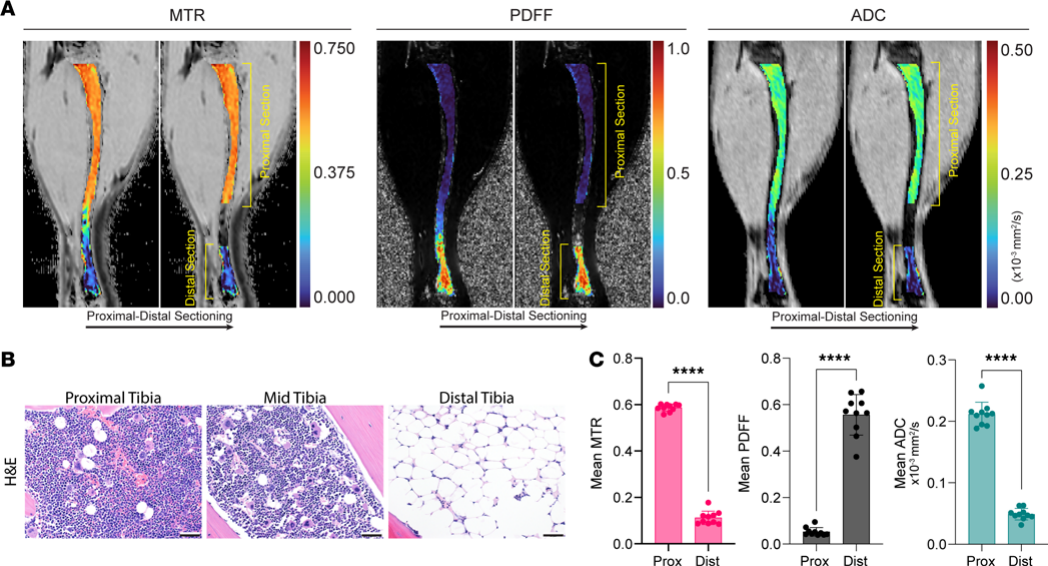
Quantitative MRI metrics identify regional differences in healthy mouse tibia BM. MRI and histology show quantitative differences in BM along the length of the tibia. (**A**) Representative pseudocolored sagittal MRI images with proximal-distal sectioning for MTR, PDFF, and ADC parameters, (**B**) representative H&E histology, and (**C**) quantification of each metric in the identified proximal and distal sections (*n* = 10 mice). Data presented as mean ± SD. *P* values calculated using Bonferroni’s multiple comparisons test. ****P < 0.0001. Histology scale bar: 50 μm.

To ultimately understand disease-related changes in the BM by MRI, we first needed to establish an MRI baseline of healthy, untouched BM. We imaged the tibias of a cohort of 10 healthy BALB/c mice, manually segmented the BM (Figure 2A), and compared each MRI metric to histology (Figure 2B). Due to consistent differences in MRI signals in the proximal and distal tibia BM, we divided the BM into “proximal” and “distal” regions (Figure 2A and Supplemental Figure 1; supplemental material available online with this article; https://doi.org/10.1172/jci.insight.161457DS1) and analyzed the MRI signal from each region separately. In healthy mice, the PDFF is consistently lower in the proximal than the distal section of the tibia, while both MTR and ADC values are higher in the proximal section (Figure 2C). These data establish that our MRI metrics capture known regional variations in hematopoietic cellularity in mouse tibia (28, 35), supporting use of quantitative MRI to analyze spatial variations in BM.

### Longitudinal changes in BM composition reflected in MRI metrics.

**Figure 3:**
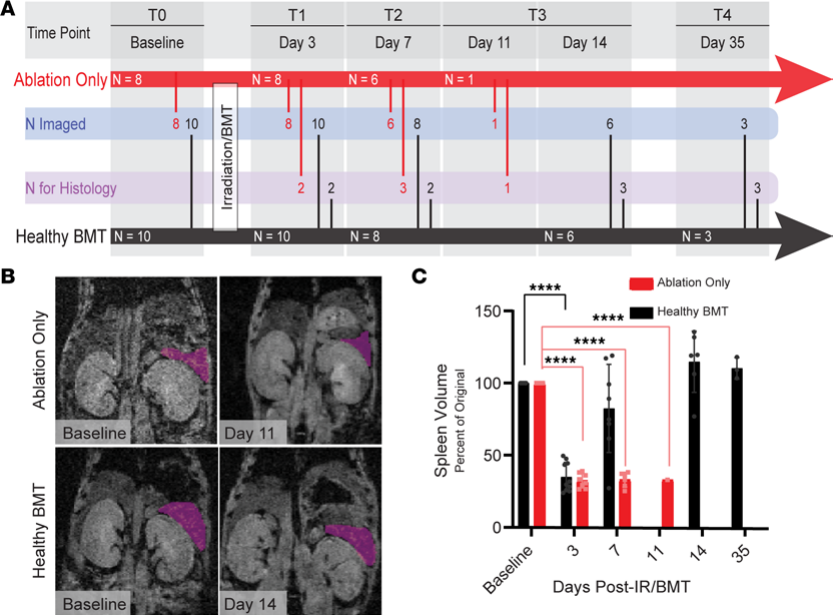
MRI quantifies changes in spleen volume in response to transplantation with healthy hematopoietic stem and progenitor cells. (**A**) Experimental timeline for ablation-only and healthy BMT, including numbers of mice remaining in each group (in arrow), numbers of mice imaged (N imaged), and numbers of mice euthanized for histology after imaging (N for Histology). Days measured post–ionizing radiation (IR)/BMT where time point zero (T0) denotes the baseline imaging of each mouse prior to IR/BMT and time points 1 through 4 (T1–T4) are after IR/BMT. The difference between the number of mice in the ablation-only group on days 7 and 11 includes 2 mice euthanized after day 7 but prior to the day 11 time point. (**B** and **C**) Changes in spleen volumes of ablation-only and healthy BMT groups. Representative coronal MRI scans with spleen highlighted in pink in **B** and volume quantification in **C**. Data presented as mean ± SD. *P* values calculated using Bonferroni’s multiple comparisons test. ****P < 0.0001.

**Figure 4:**
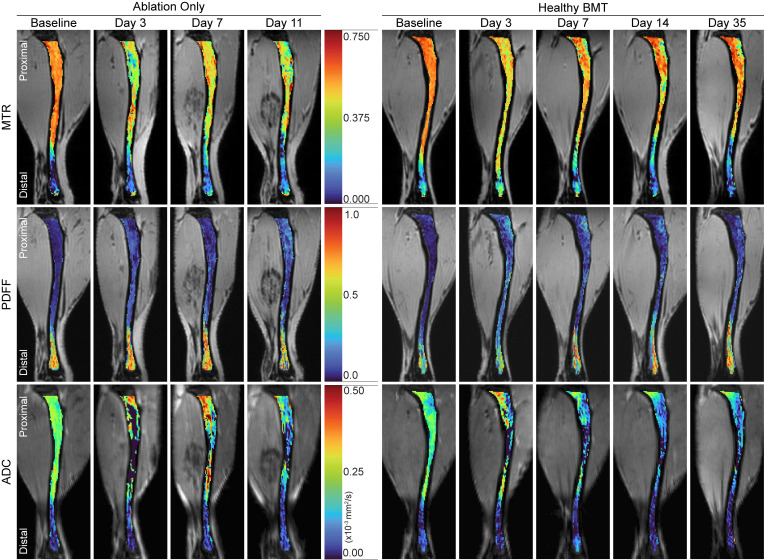
Quantitative MRI shows regional changes in BM after transplant. Representative, pseudocolored ablation-only and healthy BMT sagittal images of tibia BM showing MTR, PDFF, and ADC overlaid on corresponding, time point–matched grayscale background images (MToff; multi-gradient multi-echo mean; and low b-value, respectively). Pseudocolor scales depict range of values for each parameter. Note different ranges of values for each scale bar.

**Figure 5:**
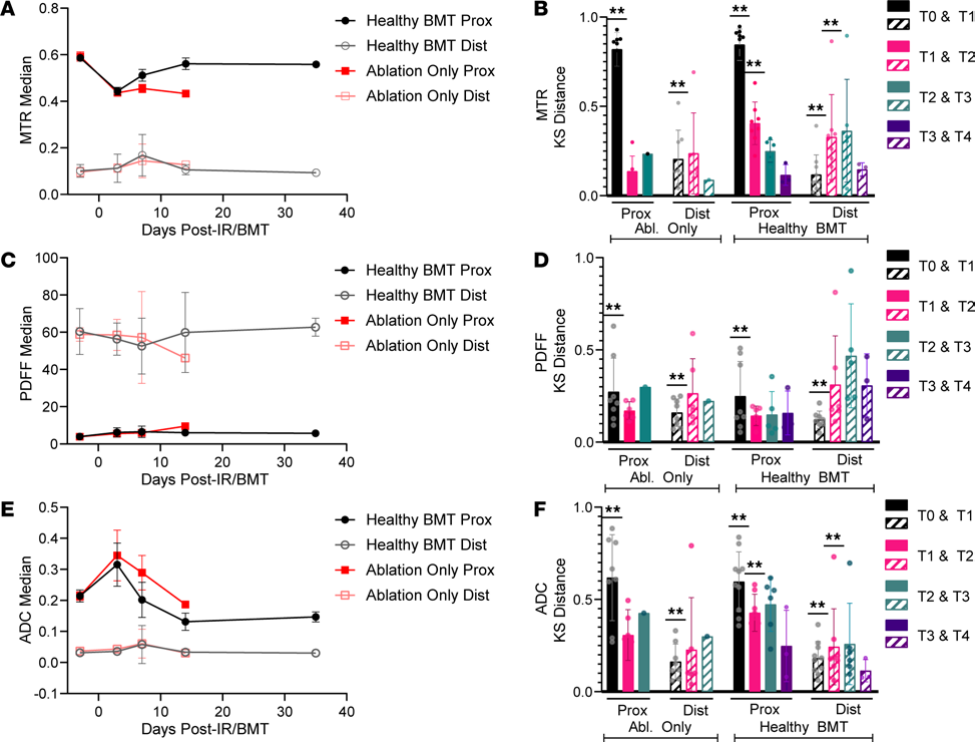
MRI metrics quantify longitudinal changes in BM after transplantation. Longitudinal changes in MTR, PDFF, and ADC for proximal and distal regions of mouse tibia BM for ablation-only and healthy BMT groups. Median trajectories (median ± SD) and longitudinal comparisons of the proximal and distal BM regions for MTR (**A** and **B**), PDFF (**C** and **D**), and ADC (**E** and **F**). We analyzed data with Kolmogorov-Smirnov (KS) distances calculated between specified time points for matched mice in **B**, **D**, and **F**. KS data presented as mean ± SD. *P* values calculated using Wilcoxon’s signed rank test with Bonferroni correction. α = 0.0125, **P < 0.0125. Histology scale bar: 50 μm. Prox, proximal; Dist, distal.

We use a transduction-transplantation mouse model of MF that first requires total body irradiation to eliminate the BM compartment, followed by transplant of HSPCs expressing a driver mutation of MF (MPL^W515L^). To separate the simultaneous effects of BM transplant (BMT) and disease progression on MRI measurements of BM, we first quantified the extent and duration of the transplant procedure itself on spleen volume and tibial BM ADC, MTR, and PDFF values. One cohort of mice received only myeloablative irradiation (ablation-only group) with mice surviving no longer than 11 days post–ionizing radiation (post-IR). The other underwent irradiation followed by transplant with healthy HSPCs (healthy BMT; Figure 3A). By day 3 post-IR/BMT, spleen volume significantly decreased by more than 50% from baseline in both the ablation-only and healthy BMT groups (Figure 3, B and C). Ablation-only spleen volumes remained low throughout the experiment, while healthy BMT spleen volumes began to recover by day 7 and recovered to approximately baseline levels by day 14. Tibia BM (Figure 4) exhibited the greatest decreases in MTR (Figure 5, A and B) and increases in ADC (Figure 5, E and F) immediately after irradiation. This likely reflects the initial loss of cellularity and cohesive macromolecular structure (decreased MTR) and reduced constraints on water movement in BM (increased ADC). We detected only modest changes in tibia PDFF (Figure 5, C and D), likely attributable to a high fat content preventing further adipocyte infiltration or expansion following irradiation (36, 37). The distal tibia showed minimal changes in MTR and ADC, which we ascribe to a higher fat content in this part of the bone (Figure 5C).

**Figure 6:**
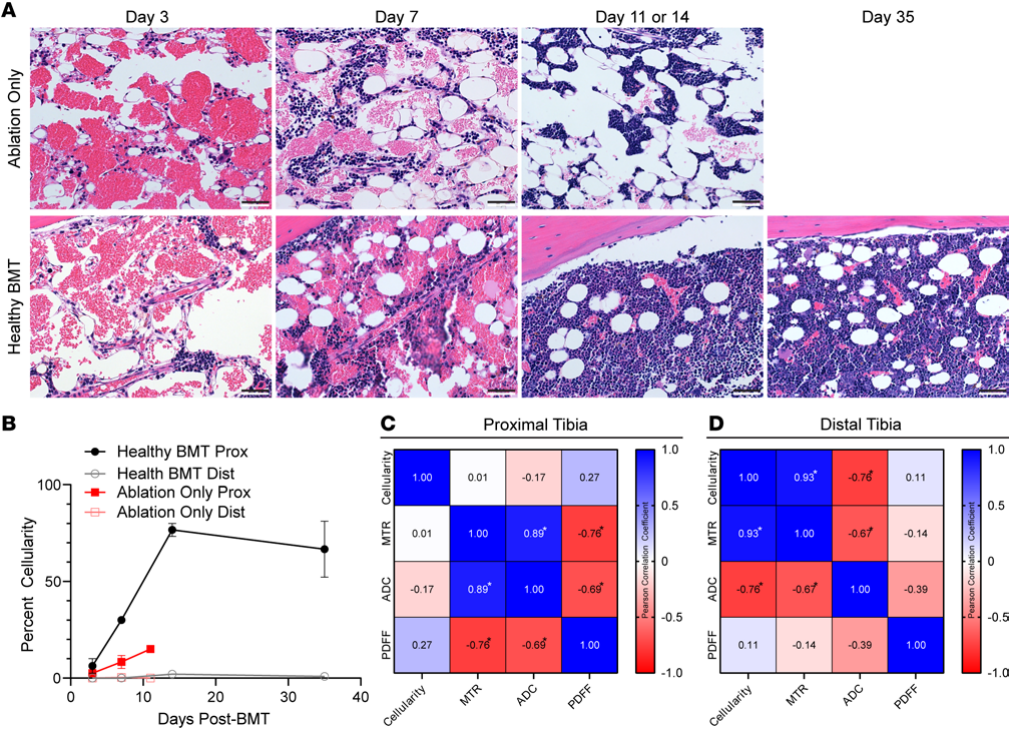
Longitudinal BM changes correlate with quantitative MRI metrics. (**A**) Representative BM histology images from ablation and healthy BMT groups stained with H&E and (**B**) quantified percent cellularity in proximal and distal parts of the tibia for each time point (number of mice at each time point in Figure 3A; data presented as mean ± SEM). Proximal (**C**) and distal (**D**) Pearson correlation matrix among cellularity, MTR, ADC, and PDFF. α = 0.01, *P < 0.01. Histology scale bar: 50 μm.

To validate changes in each MRI metric, we correlated histology with BM MRI measurements at each time point after irradiation or BMT. Ablation-only and healthy BMT groups both displayed hemorrhagic BM at early time points (Figure 6A), reflecting known damaging effects of irradiation on endothelial cells in BM vasculature (38). Following transplantation with healthy BM, cellularity in the proximal tibia consistently increased until day 14 following BMT (Figure 6B). The MTR signal correlated highly with assessed cellularity in the proximal (Figure 6C; *r* = 0.93, *P* < 0.0001) but not distal tibia (Figure 6D). ADC in the proximal tibia correlated negatively with cellularity (*r* = –0.76, *P* = 0.00072) with minimal correlation in the distal tibia (Figure 6, C and D). ADC and MTR had a negative relationship in the proximal tibia while having a positive relationship in the distal tibia. The negative relationship is most apparent at early time points post-IR/BMT (Figure 5, A and E), suggesting it results from the change from healthy to hemorrhagic BM following irradiation. Together, these results establish that the presented quantitative MRI metrics capture histologic changes in BM following irradiation and BMT. Furthermore, spleen and BM MRI demonstrate recovery to normal levels by day 14 following BMT, consistent with previous reports (39), suggesting that changes after day 14 are not the result of the transplant process.

### MRI detects changes in MPN BM and spleen volume following treatment with ruxolitinib.

**Figure 7:**
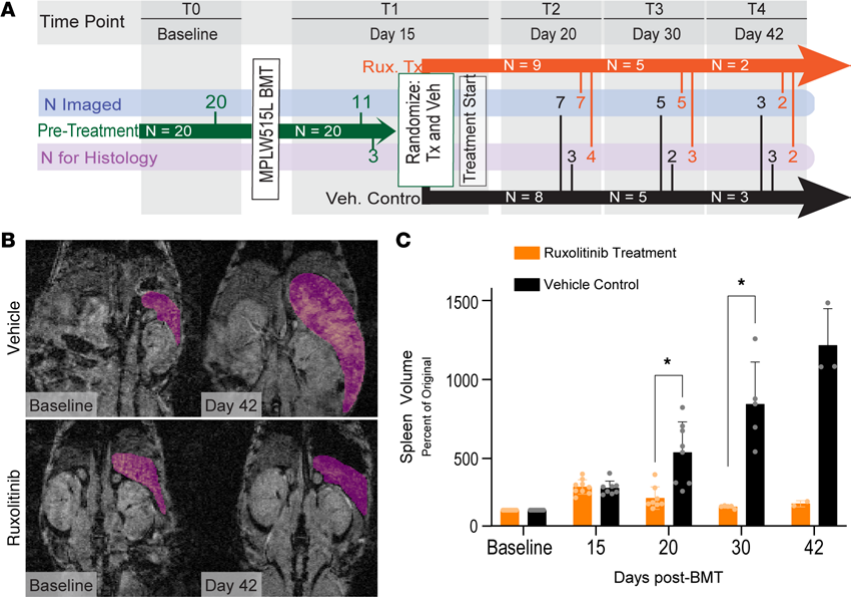
MRI monitors spleen volume in response to therapy with ruxolitinib. (**A**) Experimental timeline for ruxolitinib treatment (Rux Tx; Rux) and vehicle control (Veh. Control; Veh) groups, including numbers of mice remaining in each group (in arrow), number of mice imaged (N Imaged), and number of mice euthanized for histology after imaging (N for Histology). Days denote time after transplantation of CD117^+^ HSPCs transduced with the MPL^W515L^ mutation. We randomly assigned mice to ruxolitinib treatment or control groups after imaging on day 15 (N per group from panel **A**). (**B** and **C**) Panels show changes in spleen volumes for vehicle control and ruxolitinib treatment groups. Representative coronal MRI scans with spleen highlighted in pink in **B** and volume quantification in **C**. Data presented as mean ± SD. *P* values calculated using Bonferroni’s multiple comparisons test. *P < 0.05.

To determine the extent to which we can monitor disease progression and response to therapy in MPN/MF by MRI, we transplanted mice with HSPCs transduced with a retrovirus expressing the MPL^W515L^ driver mutation (Figure 7A) and analyzed increases in spleen volume by MRI. By day 15 after BMT, spleen volumes more than doubled from the original size, exceeding 200 mm^3^ (Figure 7, B and C). We then randomly assigned mice to treatment with ruxolitinib or vehicle. Ruxolitinib rapidly decreased spleen volumes to normal within 15 days, while vehicle control spleen volumes continued to increase significantly (Figure 7, B and C). In a separate cohort of mice, we establish that spleen volume correlates with blood counts in healthy and MPN conditions, reinforcing spleen volume as a secondary marker of MPN progression in this mouse model (Supplemental Figure 2).

**Figure 8:**
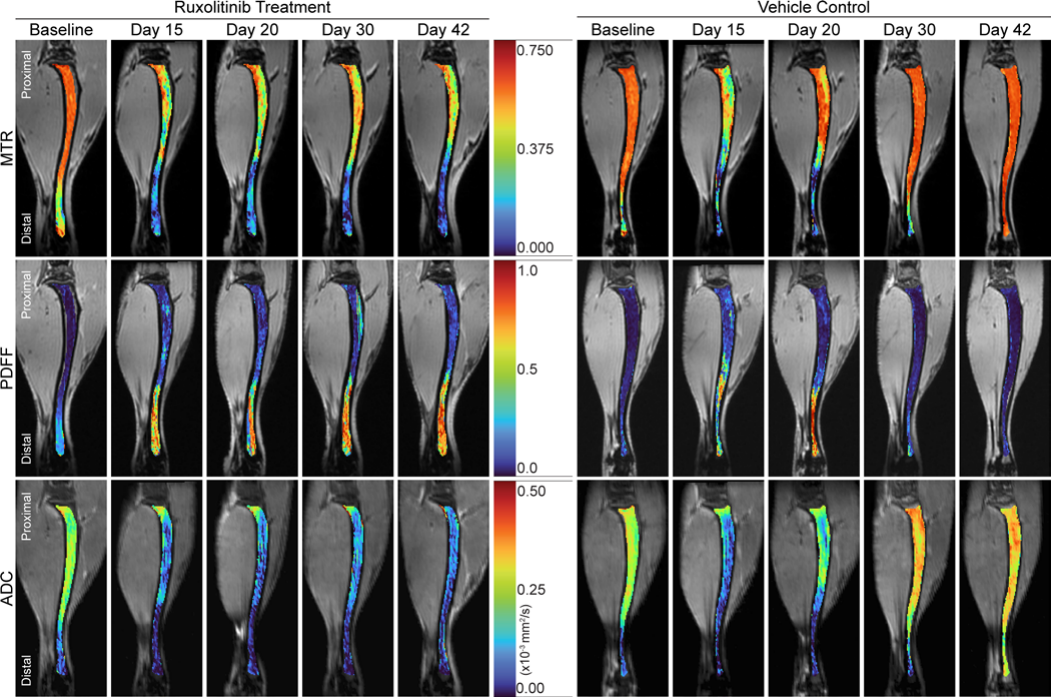
Quantitative MRI metrics show regional variations in disease progression and during treatment. Representative, pseudocolored ablation-only and healthy BMT sagittal BM slices of MTR, PDFF, and ADC overlaid on corresponding, time point–matched, nonquantitative, grayscale background images (MToff; multi-gradient multi-echo mean; and low b-value). Pseudocolor scales depict range of values for each parameter.

**Figure 9:**
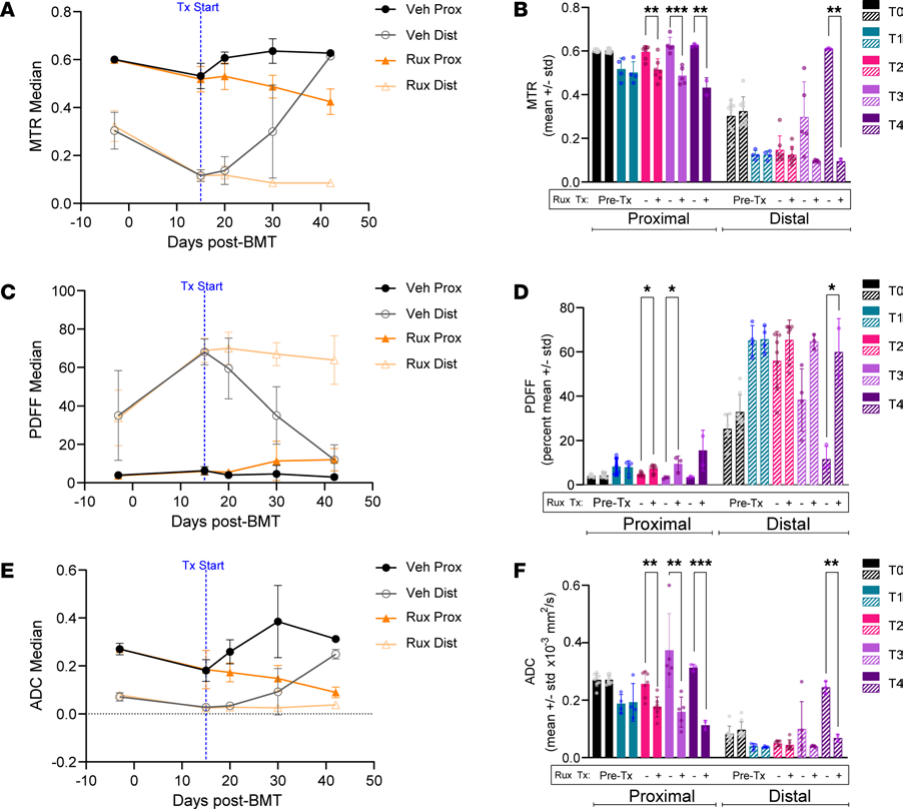
Quantitative MRI parameters detect BM response to therapy with ruxolitinib. Longitudinal changes in MTR, PDFF, and ADC in proximal and distal regions of the tibia for ruxolitinib treatment and vehicle control groups. (**A**, **C**, and **E**) Median trajectories (median ± SD) and (**B**, **D**, and **F**) comparison between treatment and control groups (mean KS distance ± SD) for proximal and distal regions of BM. *P* values calculated using Bonferroni-corrected unpaired *t* test. α = 0.02. *P < 0.02, **P < 0.01, ***P < 0.001.

We analyzed changes in tibial BM by MRI through day 42 following BMT, by which time only 3 mice remained alive in the vehicle group. At approximately this time point, data from a separate cohort of mice showed that more than 95% of all HSPCs contain the MPL^W515L^ mutation based on coexpressed GFP in the viral vector (Supplemental Figure 3). Vehicle and ruxolitinib tibia BM MRI metrics (Figure 8) significantly differed in the proximal tibia by the first time point after starting treatment (20 days after BMT) (Figure 9, B, D, and F; *P* < 0.02). Significant differences in the smaller distal tibia occurred by the final time point (day 42 after BMT; *P* < 0.02). MTR values of the vehicle control group trended higher than baseline in the proximal section, and the distal section rose significantly (*P* = 0.012 by Mann-Whitney test) to more than double the baseline value (Figure 9A). PDFF measurements decreased with a time course and distribution comparable to increases in MTR (Figure 9C). Proximal and distal tibia ADC values of vehicle control mice also trended higher than both regions in ruxolitinib-treated mice (Figure 9E).

**Figure 10:**
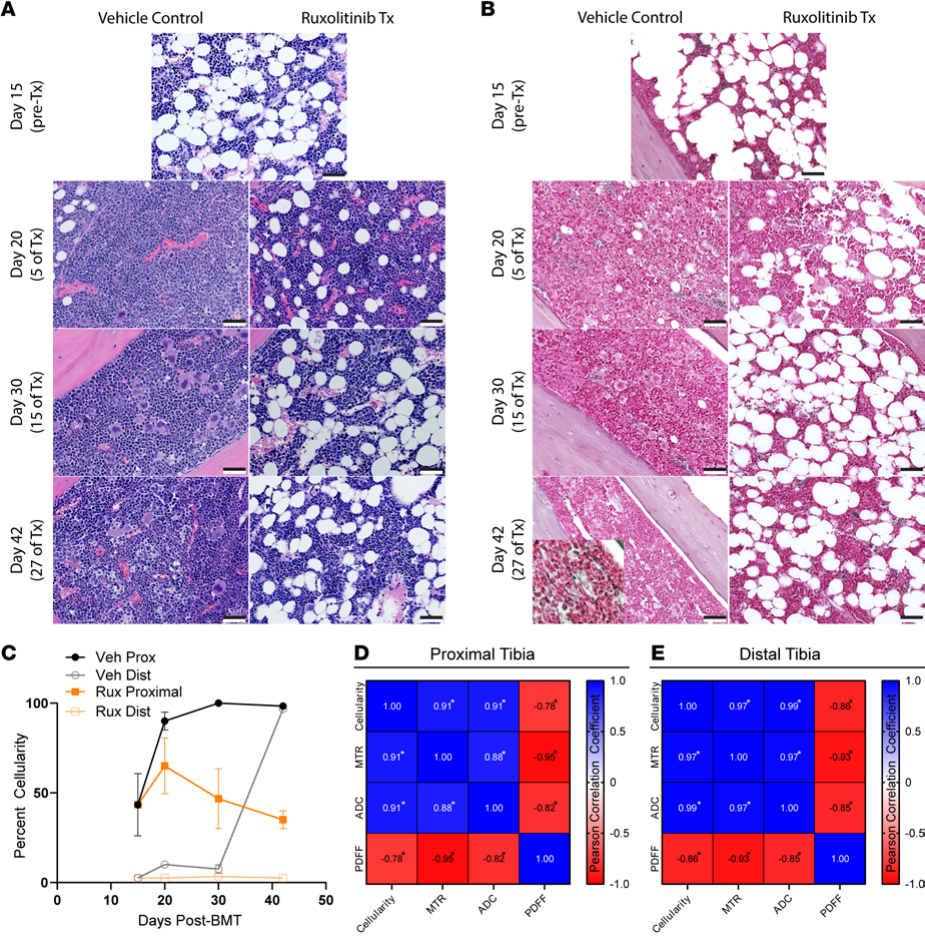
MF mouse tibia BM correlates with quantitative MRI metrics. (**A**) Representative H&E and (**B**) reticulin histology images at listed time points for each group. Day 42 vehicle reticulin insert magnified to highlight patchy fibrosis (insert contrast and saturation adjusted to better display reticulin fibers). (**C**) Graph shows changes in proximal and distal cellularity determined by a pathologist for each time point (number of mice at each time point specified in Figure 7A; data presented as mean ± SEM). (**D**) Proximal and (**E**) distal Pearson correlation matrix among cellularity, MTR, ADC, and PDFF. α = 0.01. *P < 0.01. Histology scale bar: 50 μm.

Histology from each time point (Figure 10, A and B) showed BM cellularity increased as expected across the length of the tibia in the vehicle control group (Figure 10C). Correlation with each MRI metric exhibited strong, positive relationships between MTR, ADC, and cellularity with a similar but negative relationship with PDFF (Figure 10, D and E). The inverse relation of MTR and PDFF reflects hypercellular BM replacing normal fat, a common phenomenon in MPNs overall and some patients with MF and advanced fibrosis (MF-3) (40). Reductions in PDFF corresponded with increased ADC measurements in the vehicle group. The increase in ADC with hypercellularity in BM likely occurs because of greater cellular water, elevating ADC above the very low values present in fat. Increased ADC in hypercellular BM differs from solid tumors, where greater cellularity typically reduces ADC values compared with healthy cells in surrounding tissues (41). By comparison, ruxolitinib treatment reduced ADC and preserved BM fat (Figure 9, C and E, and Figure 10A). We observed patchy reticulin fibrosis (Figure 10B) at day 42 following BMT in the proximal BM in 2 of 3 vehicle mice and no ruxolitinib-treated mice. However, the low fiber density limited our ability to effectively correlate fibrosis with MRI metrics. Overall, MRI revealed that ruxolitinib maintained BM at approximately pretreatment levels for the duration of the treatment, while the vehicle group showed progressive disease measured by all MRI parameters (Figure 9, A, C, and E). These data demonstrate that our BM MRI metrics successfully analyze disease progression and response to treatment in MPNs. The high correlation of MRI with histology further validates our methods for quantifying changes in BM composition over time and in response to treatment.

## Discussion

Monitoring BM heterogeneity at presentation and during treatment for MPNs remains an unmet need in both mouse models and clinical oncology. In preclinical models, investigators monitor disease severity and therapeutic success through blood counts, endpoint assays for spleen size, and histology of BM and other tissues. These approaches only indirectly detect response to treatment over time (blood counts) and cannot identify variations in extent of disease among subjects at the start of therapy that may impact treatment outcomes. Even with histology, investigators typically analyze limited numbers of tissue sections rather than the entire volume of BM in a site, potentially missing heterogeneous manifestations of disease within a bone. Clinical oncologists also rely on standardized scoring systems for symptoms and BM aspirates and biopsies. Clinical scoring systems determine prognosis but do not predict response to therapy (5, 42). BM aspirate/biopsy randomly sample a very limited volume of BM from the iliac crest. Biopsies suffer from sampling error, the potential for a nondiagnostic sample, and limited repeatability of this invasive, painful procedure. The relatively recent shift toward developing therapeutics that restore BM architecture for healthy hematopoiesis underscores the importance of quantitative approaches to analyze BM over time in living mice and, ultimately, patients. Our quantitative, longitudinal MRI measurements of BM and spleen provide additional information beyond existing methods, establishing a new approach to study disease mechanisms and evaluate promising drug candidates.

**Figure 11:**
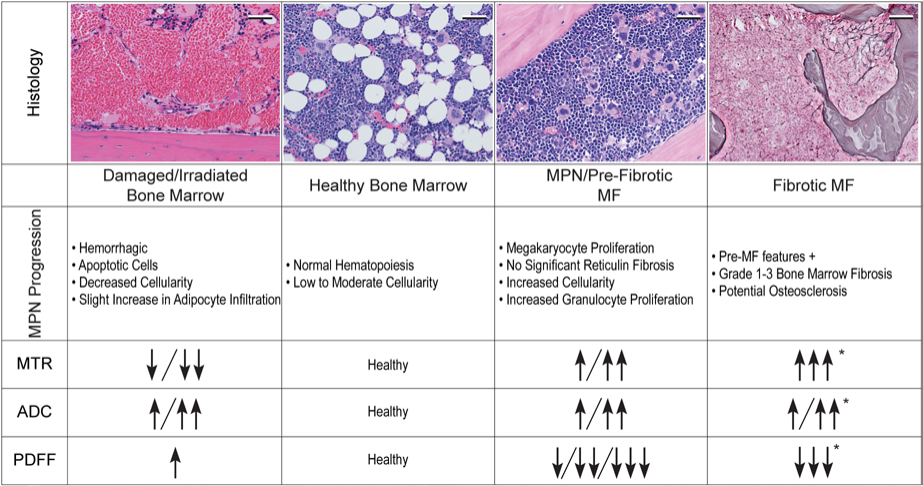
Relationship among quantitative MRI metrics and BM disease in MF. Figure highlights key changes we observed in MTR, ADC, and PDFF as they relate to various BM phenotypes. Columns show the transition from healthy BM to damaged/irradiated BM (left) or progressive MPN and eventual fibrotic MF (right). Rows represent how each metric changed according to the BM phenotype observed. Increases compared with healthy BM in the specified metric are denoted with an upward arrow (↑) while decreases are indicated with a downward arrow (↓). Multiple arrows indicate greater relative changes in the specified direction. An asterisk indicates preliminary changes of the MRI metrics in the context of fibrotic MF. The left 3 histology images are representative H&E sections of the specified BM condition. The right-hand histology is representative silver reticulin-stained histology showing moderate fibrosis in mouse BM. Histology scale bar: 50 μm.

In this study, we used clinically approved MRI metrics — ADC, MTR, and PDFF — to noninvasively analyze spatial and temporal changes in tibia BM in a mouse model of MF. With longitudinal imaging and histological validation, we demonstrated that each MRI metric detects changes occurring in BM over time following marrow ablation and HSPC transplantation. In mice transplanted with MPL^W515L^ HSPCs, MRI showed progressive increases in ADC and MTR with a reduction in PDFF, reflecting expansion of hematopoietic cells and replacement of normal fat in BM. Treatment with ruxolitinib reversed the disease phenotype toward healthy BM as quantified by decreasing ADC and MTR with greater PDFF. We also detected more pronounced changes in these parameters in the distal tibia, likely due to typically higher distal fat content in healthy mice. This result emphasizes the value of MRI to capture regional heterogeneity of disease and treatment responses. By correlating observed MRI changes with treatment, disease severity, and histological analysis, we established that imaging metrics accurately represent shifts in BM cellularity and macromolecular structure. Based on these data, we summarize how changes in each MRI measurement quantify disease progression in mouse MPN/MF (Figure 11). We also demonstrated that anatomic MRI detects changes in spleen volume, establishing a direct preclinical correlate for the current imaging standard in human clinical trials.

Matsuura et al. previously demonstrated that qualitative comparison of T2-weighted MRI identifies progressive MF with diseased BM showing higher signal than control (29). However, there are multiple potential causes of increased T2 signal in BM, making this a very a nonspecific imaging finding (43). While qualitative MRI excels at identifying variations in tissue morphology, such as visualizing solid tumors, determining increased signal intensity in a site requires comparison with normal tissue and/or changes over time. Consequently, clinical application of qualitative T2-weighted BM imaging is limited because patients rarely have healthy BM MRI scans for comparison. Qualitative assessments of signal intensity lack standardization and suffer from inter-reader variability, severely limiting the reproducibility needed to monitor variations in disease progression and treatment. To overcome these limitations and increase future applications of BM MRI in multicenter clinical trials and clinical oncology, we use multiple quantitative MRI metrics to longitudinally assess BM in MF. Although ADC, MTR, and PDFF measure nominally distinct parameters, interdependence among biophysical and chemical factors in BM makes results from a single metric challenging to interpret. As summarized in Figure 11, we show that ADC increases both with cellularity in progressive MPN and release of intracellular water during cell death after irradiation. MTR increases with both cellularity and fibrosis, making it difficult to distinguish between the 2 by MTR alone. MTR and ADC of adipocytes are very low, so progressive loss of BM fat likely leads to increases in each parameter. Combining increased ADC and PDFF with decreased MTR indicates the regime of cell death post-IR before BM reconstitution. Concordant changes in MTR and ADC suggest progression or regression of BM hypercellularity and fibrosis. Since each MRI metric is not wholly independent of the others, combining parameters as we describe provides a more accurate overall assessment of disease status and response to therapy. Furthermore, the quantitative nature of these metrics ensures that measurements from a single time point can be placed along a disease continuum, providing immediate information on disease severity and the impact of treatment.

The quantitative MRI metrics used in this study have been used previously in various other diseases characterized by fibrosis or changes in fat content. ADC can reduce the need for biopsy in cases of suspected liver cirrhosis (44), while MTR distinguishes healthy versus postradiation fibrotic tissue in rectal cancer (45). PDFF decreases with fibrotic progression in fatty liver disease (46). Quantitative measurements of fat content are used routinely in clinical medicine to monitor steatosis in the liver (47), and multiparametric MRI (including MTR) has successfully monitored development of fibrosis in folic acid-induced nephropathy in mice (48). These studies underscore the feasibility of implementing quantitative MRI methods in preclinical and clinical evaluation of MPNs such as MF.

Due to the aggressiveness of the MPL^W515L^ HSPC transplantation model for MF, individual mice often did not survive long enough to develop pronounced BM fibrosis. Therefore, additional studies are needed to validate our promising initial data showing that multiparametric, quantitative MRI may detect BM fibrosis. Measuring changes in BM fibrosis with MRI will provide investigators a longitudinal, noninvasive method to evaluate the next generation of drug candidates focused on reversing fibrosis and restoring the BM environment in MF. Furthermore, past studies establish that mice respond much more positively and uniformly to ruxolitinib treatment than patients (17, 49). As a result, our imaging study detects clear differences between ruxolitinib treatment and control groups nearly immediately after initiating therapy. Because BM adipose tissue tends to increase with age and tends to be higher in males compared with females (50–53), we utilized only 6- to 8-week-old female mice for consistency among cohorts. However, since MPNs and MF occur in older men and women, further studies are required to investigate the possibility for age and/or sex-related differences in disease progression and therapeutic response.

Determining to what extent quantitative BM MRI can reduce the need for BM aspiration/biopsy in patients represents an important future direction for our work. While we do not anticipate that quantitative BM MRI will eliminate the need for biopsy altogether, this imaging method could improve conclusions from a biopsy. For example, imaging might reveal to what extent a biopsy captures macroscopic features of the wider BM compartment. MRI also analyzes a much larger volume of BM, detecting heterogeneity of disease within a bone or among multiple different bones. Because imaging reveals spatial heterogeneity of disease in the BM, MRI could in principle be used to identify regions of interest for biopsies. However, given the routine nature of BM biopsies and logistics of the procedure, we do not anticipate widespread use of image-guided BM biopsies for MF.

Overall, we demonstrate a robust MRI framework for noninvasively evaluating BM during disease progression and treatment in mouse MPNs and more specifically in MF. Our imaging strategy detects heterogeneity of BM as validated by histopathology over time, establishing a powerful new noninvasive approach to investigate mechanisms of disease progression and test promising new therapies in MPNs and other diseases that manifest in BM. Because ADC, MTR, and PDFF are approved for MRI in humans, the current study sets the stage for coclinical trials of treatments for MF, building on our past research using PDFF to detect variable responses to ruxolitinib in patients with MF (23). Ultimately, we envision the potential to validate quantitative BM MRI as a biomarker for staging disease severity and predicting early response to therapy in MF. While the current study focused on MF, we anticipate that these or similar quantitative multiparametric MRI methods could readily be applied to analyze BM in other hematologic malignancies.

## Methods

### Retrovirus vectors and cell culture.

We maintained HEK293T cells from the American Type Culture Collection in DMEM (catalog 10313, Gibco, Thermo Fisher Scientific) supplemented with 10% FBS (HyClone, Thermo Fisher Scientific), 1% GlutaMAX (catalog 35050, Gibco, Thermo Fisher Scientific), 50 mg/L Plasmocin prophylactic (InvivoGen), and 1% Penicillin-Streptomycin (catalog 15140, Gibco, Thermo Fisher Scientific). We transfected HEK293T cells (3.5 × 10^6^ cells seeded 1 day before) by calcium phosphate precipitation (54) with 10 μg per plate each of MSCV-MPL^W515L^-IRES-EGFP (55) retroviral vector (gift of Ann Mullally, Dana-Farber Cancer Institute, Boston, Massachusetts, USA) and psiEco packaging plasmid. We recovered cell culture supernatants with MPL^W515L^ retrovirus 2 days after transfection.

### Mouse model and BM transplant.

We housed mice as previously described (56). We purchased adult (6- to 8-week-old), female, BALB/c mice from Charles River Laboratories and transplanted retrovirally transduced HSPCs as previously described (7). Briefly, we enriched donor BM for CD117-positive cells via positive selection with MACS microbeads (catalog 130-091-224, Miltenyi Biotec). We transduced enriched cells in transplant medium (RPMI 1640 + 10% FBS; 6 ng/mL IL-3, 10 ng/mL IL-6, and 10 ng/mL stem cell factor) in a plate coated with RetroNectin (catalog T100B, Takara Bio) using retroviral supernatants (1 mL supernatant per 1 × 10^6^ cells) with 5 μg/mL polybrene (Sigma-Aldrich) via spin infection (18,700*g*, 90 minutes at room temperature). We removed viral supernatants from cells after 6 hours, cultured overnight, and then intravenously transplanted 5 × 10^5^ cells (1:1 mixture of CD117-enriched cells with non-CD117 BM cells) via tail vein injection into sublethally irradiated recipients (2 × 4.5 Gy separated by 24 hours). We euthanized animals for histology at prespecified time points or when moribund. For mice receiving healthy BM transplants, we cultured CD117-enriched cells overnight without retroviral transduction and then transplanted similarly.

For complete blood counts (CBCs), we collected blood into K^2+^ EDTA anticoagulant tubes from the submandibular vein of healthy BALB/C mice or from mice transplanted with MPL^W515L^-transduced HSPCs. Diseased blood was obtained at days 25 and 45 after BMT. We submitted samples to the University of Michigan In-Vivo Animal Core for analysis on a Heska Element HT5 automated veterinary hematology analyzer.

We recovered BM from femur and tibia of mice 45 days after transplanting with MPL^W515L^GFP^+^-transduced HSPCs. We lysed red blood cells and stained for Lin^–^Sca-1^+^c-Kit^+^ (LSK) for HSPCs. We used the following Abs purchased from Thermo Fisher Scientific to identify LSK cells: Cd16/32 Fc block; biotinylated anti-CD3e (clone 145-2C11), Gr-1 (clone RB6-8C5), B220 (clone RA3-6B2), Ter119 (clone TER-119), CD11b (clone M1/70), and CD11c (clone N418) with Pacific Orange–conjugated streptavidin to exclude differentiated lineages; PE-conjugated anti-CD117 (clone 2B8; c-kit); and APC-conjugated anti–Sca-1 (clone D7). We obtained percentage GFP^+^ HSPCs by flow cytometry using FCS Express 7 Research Edition.

### Ruxolitinib treatment.

We reconstituted ruxolitinib (catalog 11609, Cayman Chemical) in an 80 mM acetic acid solution of pH 4 and then added 1:1 diluted hydrochloric acid dropwise to bring the drug into solution. Once in solution, we adjusted the pH to 3.7–4.0 using sodium hydroxide and stored at 4°C for no more than 1 week. We initiated treatment with ruxolitinib (90 mg/kg twice a day) (49) or vehicle (80 mM acetic acid at pH 4) by oral gavage when the average spleen volume of the cohort reached 200 mm^3^ as measured by MRI. Treatment continued for the duration of the study. We euthanized mice for histology at prespecified time points or when moribund as measured from the start of treatment, at days 0, 5, 15, and 28.

### Histopathology.

We fixed tissues in 10% neutral-buffered formalin for at least 72 hours, dehydrated in ethanol, and submitted to the University of Michigan In-Vivo Animal Core for routine processing, paraffin-embedding, sectioning, and H&E staining. For bone samples, we performed decalcification in 0.5 M EDTA (catalog BMA51234, Lonza) according to the manufacturer’s instruction before processing. A board-certified hematopathologist independently performed histologic evaluation of tissue sections. All cases with disease involvement were confirmed by the presence of atypical megakaryocytic proliferation and marked extramedullary hematopoiesis in spleens and livers. We estimated marrow cellularity from 1 H&E slide at 5% increments in the entire tibia BM and then again separately in the proximal and distal tibia. In cases where assessed cellularity was less than 5% but with appreciable hematopoiesis, we used 2.5% for statistical analysis. BM sections with limited tissue retention were excluded from analysis. We evaluated reticulin stains performed on the BM sections by Histoserv, according to the 2016 WHO classification (57). For correlation with MRI metrics, histology samples were visually aligned with MTR, ADC, and PDFF images (Supplemental Figure 1).

### MRI of murine spleen and tibia.

We imaged mice on a 7 T 30 cm bore preclinical MRI system (Bruker BioSpec, Paravision version 7.0.0) using an 86 mm inner diameter radiofrequency (RF) transmit/receive coil for spleen imaging and a 4-channel cryogenically cooled surface coil for imaging the mouse tibia. To ensure consistent positioning within the coil, we immobilized each mouse’s right hind limb in a custom, 3D-printed support structure. For DWI, we used a standard Stejskal-Tanner (58) multislice spin-echo sequence (repetition time/echo time, TR/TE = 2,000/22 ms) with fat suppression and diffusion sensitization applied along 3-orthogonal axes at b value = 0 and 3,000 s/mm^2^ (59). For MTR imaging, we selected a 3D FLASH experiment from available vendor-provided (Bruker) sequences. We used a 3-dimensional gradient-echo sequence (TR/TE/flip = 111/3 ms/9°) without (magnetization transfer off, MToff) and with application (MTon) of 8 μT amplitude, 100 ms duration 2,400 Hz off-resonance RF saturation pulse. The MT pulse was gaussian shaped, 100 ms in duration, with an 8 μT peak amplitude demonstrated to have negligible direct saturation on water (MTRwater = –0.018 ± 0.028) compared with lamellar liquid crystal (llc) phantom that exhibits strong MT contrast (MTRllc = +0.799 ± 0.021). For PDFF imaging, we used 3D multi-echo, gradient-echo (TR = 50 ms; TE = 1.47 + [*n* × 0.317] ms where *n* = 0, 1,…11) sequence acquired over 4 series, 3 echos each. Acquisition geometry was fixed across DWI, MTR, and PDFF scans with field of view (23 mm × 9.6 mm × 6 mm) at nominal 0.1–0.18 mm spatial resolution.

### Quantitative map generation.

We reconstructed all images on the MRI system using product software and then transferred them offline for quantitative map generation using custom software developed within MATLAB version 2019b. We calculated ADC maps using the well-established mono-exponential signal decay model as a function of b value (59). For MTR, we used (MToff-MTon)/MToff to calculate maps. We reduced the 12 complex-valued gradient-echo images using a 2-dimensional graph-cut algorithm to derive PDFF maps (60).

### Image registration and volume of interest definition.

We used in-house MATLAB scripts to visualize MR images and manually segment voxels within the tibia BM. To facilitate longitudinal inspection of images in a common frame of reference, we spatially registered MRI scans of each individual mouse over multiple scan dates to the baseline time point using a rigid-body transformation with *Elastix* software (61, 62). We masked the transformation to optimize alignment of voxels within, and immediately adjacent to, the tibia volume of interest.

### Proximal and distal tibia section development.

We developed axial mean trajectories for each MRI metric by averaging axial voxel values at every image slice along the length of the tibia BM. We defined proximal and distal regions by averaging the 2 greatest points of change, identified using the MATLAB built-in function ischange, of each of the baseline ADC, MTR, and PDFF images. We then applied the proximal and distal sections to subsequent time points and extracted the corresponding region data for analysis (Supplemental Figure 1). For representative tibia images, we chose a single sagittal slice containing the entire length of tibia BM for display. For quantitative analysis and comparison of proximal and distal regions, we utilized the entire proximal or distal image volume rather than a single sagittal slice.

### Statistics.

We calculated proximal and distal tibia BM MRI metrics and spleen volume *P* values using Bonferroni’s multiple comparisons test. For 2-sample KS distances and *P* values for KS comparisons, we used MATLAB function kstest2 and the Wilcoxon’s signed rank test with Bonferroni’s correction. We used Bonferroni-corrected unpaired 2-tailed *t* test to compare treatment and vehicle control values. Unless otherwise stated, we used GraphPad Prism 9 for Pearson correlation matrices and all other statistical tests. We considered *P* values less than 0.05 to be statistically significant unless otherwise stated.

### Study approval.

The University of Michigan Institutional Animal Care and Use Committee approved all experimental procedures involving animals, and we housed mice as previously described (56).

## Author contributions

THR designed and performed research, analyzed data, and wrote the paper; MS, GA, WYL, and AZ analyzed data; BAH provided reagents; KH, JMB, and AB performed research; KP and MT contributed intellectually about biology and therapy of MF; KEL contributed to image analysis methods; DM, TLC, and BDR designed experiments and imaging methods; GDL designed experiments and wrote the paper; and all authors reviewed and edited the paper.

## Supplementary Material

Supplemental data

## Version 1 08/23/2022

In-Press Preview

## Version 2 10/10/2022

Electronic publication
