# Supplementary material for: Multiparametric MRI to quantify disease and treatment response in mice with myeloproliferative neoplasms
Source: JCI Insight. 2022 Oct 10;7(19):e161457. doi: 10.1172/jci.insight.161457 (PMC9675444; doi:10.1172/jci.insight.161457)
Supplement: Supplemental data [file jciinsight-7-161457-s032.pdf]

Supplemental Figures and Legends

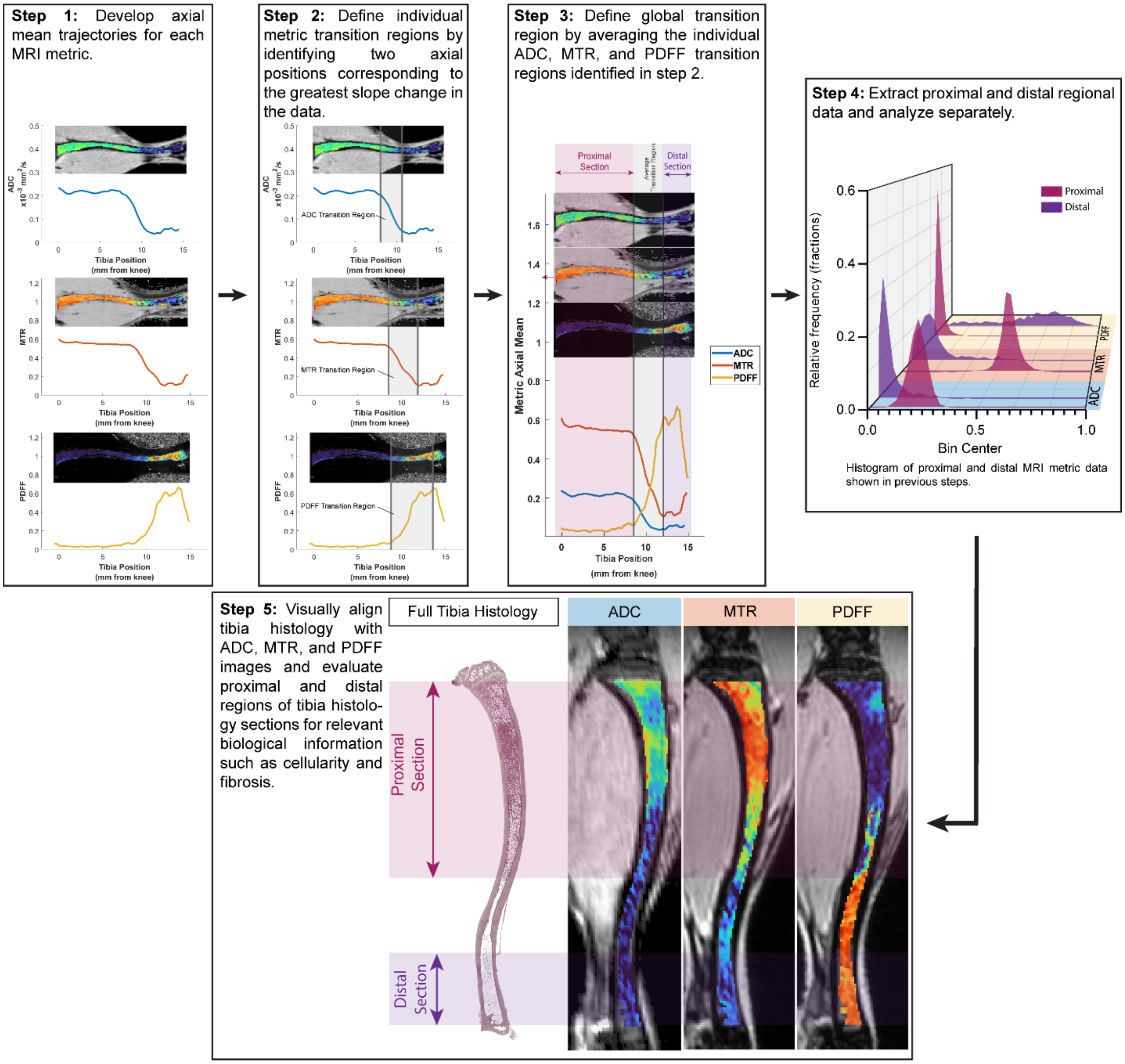

Supplemental Figure 1. **Developing proximal and distal sections in tibia MRIs and aligning to histology.** To better quantify the heterogeneity we observed along the length of mouse tibia bone marrow, we developed a proximal-distal sectioning process in order to analyze those regions separately. The first step is developing axial mean trajectories for each MRI metric. To do this, we average the voxel values of the axial dimension together and plot them along the length of the tibia (as measured by millimeters from the knee). Step 2 is identifying the transition region for each metric where the proximal section transitions to the distal section. We do this using the MATLAB function 'ischange' which enables us to identify the two points in a set of data which correspond to the greatest change in slope of the data. Step 3 is developing one transition region for all three MRI metrics by averaging together the three transition regions from the previous step. Step 4 is applying the newly developed proximal and distal regions to the images of each time point for a given mouse and then analyzing the proximal and distal regions separately. Lastly, in Step 5, we visually align the proximal and distal regions of the ADC, MTR, and PDFF images to the tibia bone marrow histology for validation.

Supplemental Figures and Legends

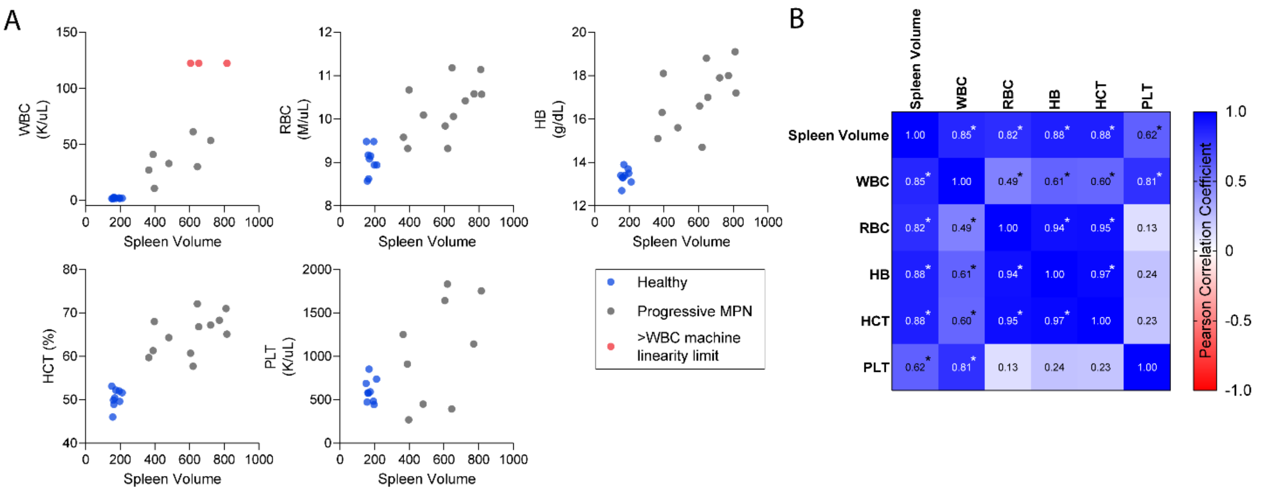

Supplemental Figure 2. Blood counts of healthy and diseased mice correlate with spleen volume. Relationship of white blood cells (WBC), red blood cells (RBC), hemoglobin (HB), hematocrit (HCT), and platelets (PLT) with spleen volume (A) and Pearson correlation (B). In scatter plots, blue point denotes healthy, gray denotes developing progressive MPN, and red denotes samples with WBC counts higher than the linearity detection limit of the analysis machine. *P<0.05.

Supplemental Figures and Legends

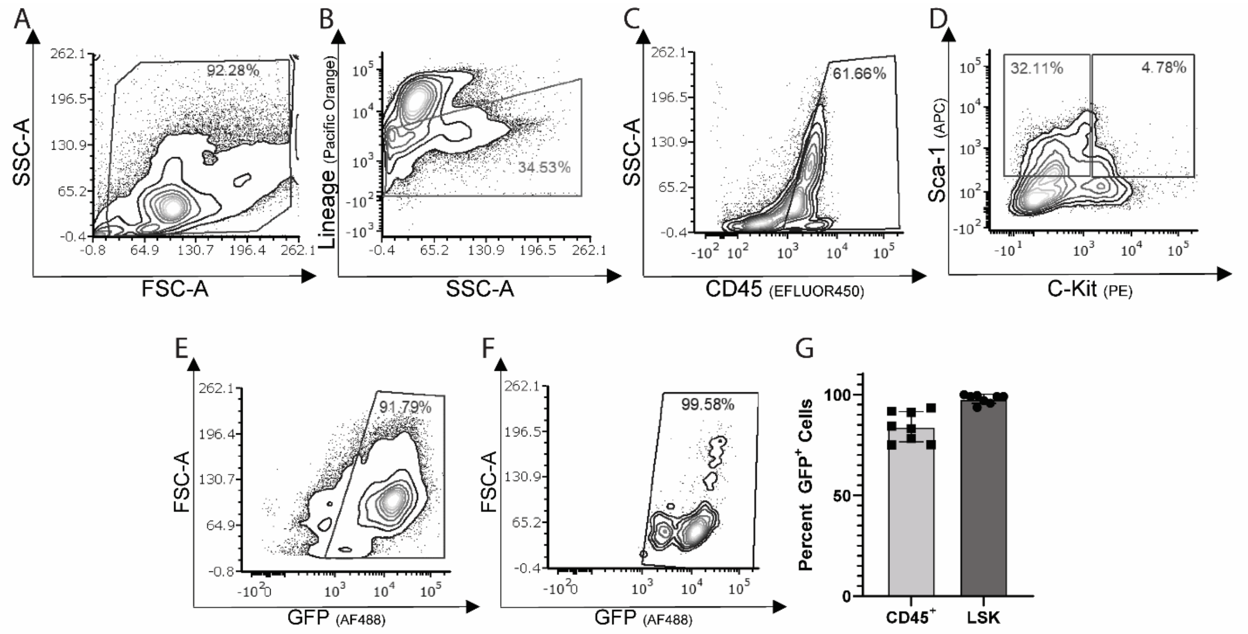

Supplemental Figure 3. Mouse bone marrow HSPCs derived primarily from MPLW515L transplanted cells. Representative flow cytometry contour plots and gating for GFP⁺ Lin⁻-Sca-1⁺-c-Kit⁺ bone marrow cells harvested from mice 45 days post-BMT with cells transduced to coexpress MPLW515L and GFP (n=8 mice). Total bone marrow cells (A) were stained for lineage markers CD3, Gr-1, B220, Ter119, CD11b, and CD11c and gated on the lineage negative population (B). CD45⁺ cells were selected (C) and gated for Sca-1⁺ and c-Kit⁺ cells (D, right gate). We report GFP⁺ cells from total Lin⁻-CD45⁺ cells (E) and LSK cells (F) with quantification (G). Data presented as mean ± SD.
